# The Metacognitive Enhancement Triad model enhances learning outcomes in first-year emergency medicine residents

**DOI:** 10.3389/fmed.2025.1720071

**Published:** 2025-11-17

**Authors:** Wu Ding, Libing Jiang, Anyu Qian, Shanxiang Xu

**Affiliations:** Emergency Medicine Department, Second Affiliated Hospital of Zhejiang University School of Medicine, Hangzhou, Zhejiang, China

**Keywords:** standardized residency training, metacognition, National Medical Licensing Examination, peer-assisted learning, problem-based learning

## Abstract

**Introduction:**

This study evaluated the Metacognitive Enhancement Triad (MET) model, integrating peer-assisted learning (PAL), problem-based learning (PBL), and practice-enhanced cognitive learning (PCL), for improving first-year residents’ learning outcomes and National Medical Licensing Examination (NMLE) performance.

**Methods:**

A quasi-experimental study was conducted at a tertiary academic medical center. 24 first-year residents received conventional training (pre-intervention), while 22 received the MET intervention (post-intervention). The MET model comprised: (1) PAL groups formed by diagnostic assessment; (2) Constructivist PBL sessions led by mentors; (3) Clinical rotations restructured to align with NMLE content (PCL). Primary outcomes were NMLE scores and pass rates. Secondary outcomes were self-reported competencies and satisfaction measured on a 10-point Likert scale.

**Results:**

The post-intervention cohort showed significantly higher NMLE scores (429 ± 41 vs. 388 ± 46, *p* < 0.01) and pass rates (90.9% vs. 62.5%, *p* < 0.01). They also reported greater improvement in professionalism, integrated clinical proficiency, lifelong learning, and overall satisfaction (all *p* < 0.05).

**Conclusion:**

The MET model significantly enhanced NMLE performance and key competencies among first-year emergency medicine residents, offering an effective framework for residency training.

## 1 Introduction

Residency training marks the beginning of a Chinese medical student’s career as a doctor (1). The first year forms the foundation of the entire training program. During this time, residents are required to pass the National Medical Licensing Examination (NMLE), which is an important indicator of the effectiveness of teaching first-year residents at training bases. The NMLE is an entrance examination for the medical profession, as well as being a necessary condition for the final assessment of standardized training for Chinese residents. The first-attempt pass rate for the NMLE is also one of the evaluation criteria for residency training bases. Helping residents complete their career transition and pass the NMLE successfully is the first problem residency training bases face (2).

In China, variability in the backgrounds of resident physicians—such as medical schools, academic degrees, specialized disciplines, and prior experience—is a significant predictor of disparities in NMLE performance (3, 4). In the past 5 years, the national pass rate for the NMLE has fluctuated around 50%, while the average national pass rate at standardized residency training bases (clinical medicine major) was approximately 79%. There are significant variations in the pass rates of the NMLE across different specialties. Compared to specialties such as internal medicine and neurology, the pass rate for the NMLE at many emergency medicine residency training bases is generally unsatisfactory (80 vs. 68%). Therefore, for many training bases, improving the pass rate of NMLE has become a pressing priority that requires immediate resolution (2).

Our graduate residents (who are also enrolled in a Master’s program in clinical medicine) maintain a 100% first-attempt pass rate year-round, but residents without graduate degrees (undergraduate residents not enrolled in a graduate degree program, mostly from primary healthcare institutions) did not perform as well. Based on our teaching observations of residents’ performance in both clinical work and academic learning, we have identified that undergraduate residents often exhibit low learning efficiency and suboptimal learning outcomes. Further analysis revealed that the underlying cause of this phenomenon is their inability to construct a systematic knowledge framework, coupled with a failure to enhance their understanding and application of theoretical knowledge through integrating it with clinical practice. These findings indicated a deficiency in their metacognitive skills. Metacognitive skill is the ability to master learning methods and strategies, enabling individuals to learn and adapt to new tasks more efficiently (5). Insufficient metacognitive skills adversely affect both success on the NMLE and impede the subsequent professional growth of resident physicians. Based on our observations of undergraduate residents, we believe that enhancing their metacognitive skills is the most fundamental solution for improving both their licensing examination performance and overall training outcomes. Unlike other studies that focus primarily on directly boosting exam scores (6, 7), our teaching methodology is more concerned with addressing the root problem observed in undergraduate residents during training—namely, the lack of metacognitive abilities. This study aims to develop a novel and systematic teaching methodology focused on enhancing residents’ metacognitive learning abilities, known as the Metacognitive Enhancement Triad (MET) model. This educational system is designed to be applicable to the broad population of residents from diverse educational backgrounds, particularly undergraduate residents. By improving the metacognitive learning skills of these residents, it will thereby increase the pass rate of the NMLE and enhance the overall outcomes of standardized residency training (8).

## 2 Methods

### 2.1 Study population

The subjects of the study were first-year undergraduate residents before and after the implementation of MET model at our residency training base.

### 2.2 MET model

**Figure 1:**
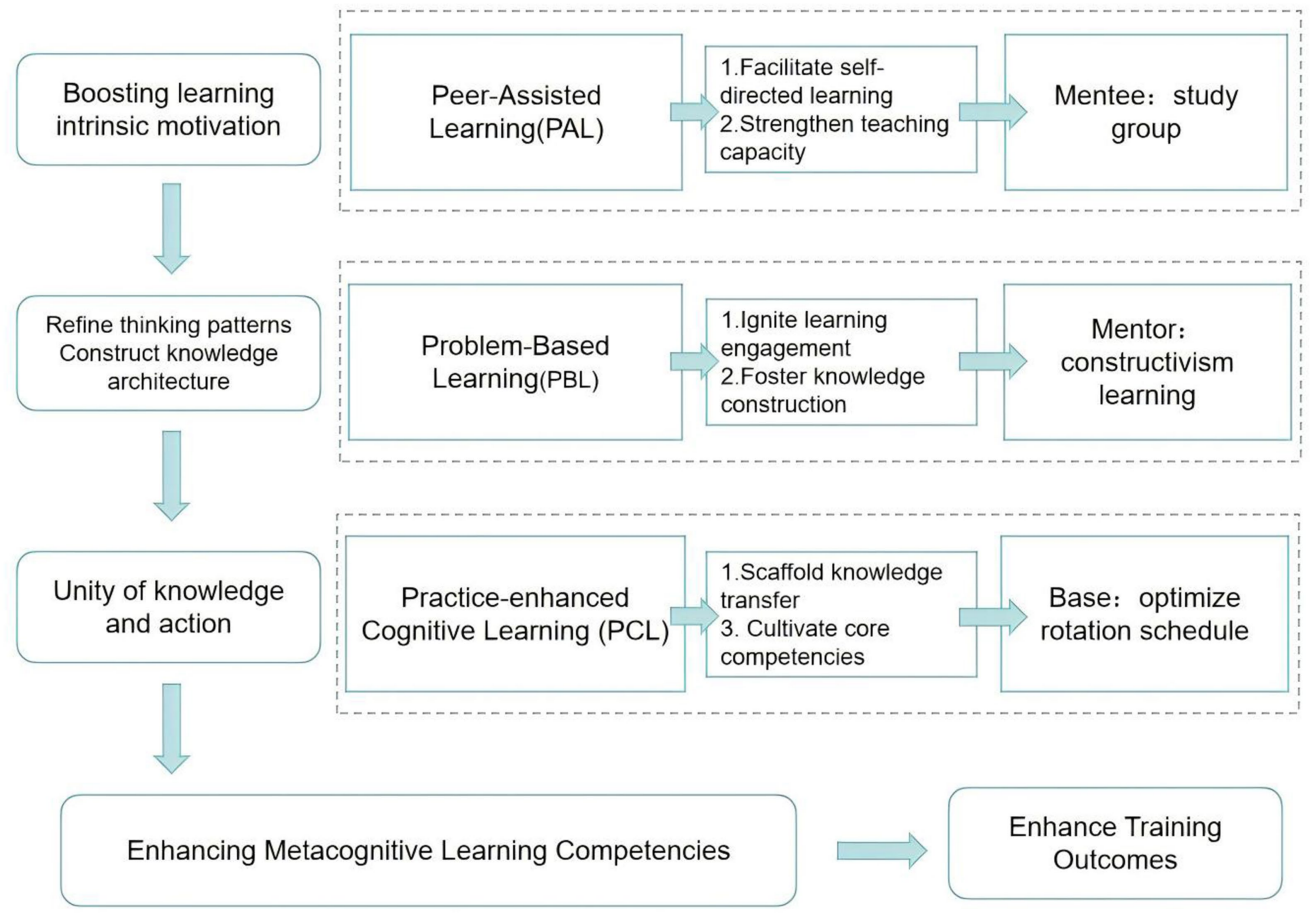
MET model to improve residents’ metacognitive skills.

To improve residents’ metacognitive skills and the learning outcomes of training, we utilized Peer-Assisted Learning (PAL), Problem-Based Learning (PBL) and Practice-Enhanced Cognitive Learning (PCL). See Figure 1 for the specific measures.

Residents-PAL: Based on the diagnostic exam scores, undergraduate and postgraduate residents were mixed into study groups of four to five residents. These groups regularly met at weekends and in the evenings to study together. The PAL groups engaged in a self-directed process centered on clinical cases encountered in practice and challenging questions arising during NMLE preparation. Participants independently researched relevant materials and develop preliminary solutions before convening for structured group discussions aimed at building initial consensus. Following each session, the group collectively determined the topic for the subsequent meeting. The postgraduate residents, with their more advanced learning experience and strategies, often acted as informal peer leaders, helping to guide discussions, share effective learning methods, and model structured problem-solving approaches. This collaborative learning and discussion format helps stimulate intrinsic motivation among undergraduate residents. Through the process, undergraduate residents were exposed to and gradually internalize the learning strategies and cognitive approaches typically employed at the graduate level. This exposure not only facilitated knowledge acquisition but also enhanced their metacognitive awareness and self-regulated learning capabilities.
Mentor-PBL: Guided by the Problem-Based Learning (PBL) approach, the instructor implemented a structured educational process involving both pre-session guidance and post-session review. During the pre-session phase, the instructor assisted residents in retrieving relevant literature, analyzing real clinical cases related to actual practice challenges, and developing appropriate solutions. Additionally, for difficult questions encountered during exam preparation, the instructor facilitated the construction of problem-solving pathways and guided residents in selecting correct answers. In the post-session review phase, the instructor actively participated in PAL-group discussions, leading systematic reflections on clinical cases to help residents synthesize practical experiences and identify lessons learned. Furthermore, incorrect or challenging test items were analyzed to clarify reasoning and address knowledge gaps. Throughout the instructional process, the focus extended beyond knowledge transmission to emphasize the application and transfer of knowledge. Grounded in constructivist learning theory (9), the instructor helped residents establish connections between prior and new knowledge, fostering the development of an integrated knowledge framework. By strengthening the linkage between theoretical knowledge and practical contexts—such as clinical work and examination scenarios—the instructor promoted deeper comprehension and mastery of the subject matter. Moreover, special emphasis was placed on stimulating intrinsic motivation and cultivating metacognitive skills, thereby supporting the development of self-directed and lifelong learning capabilities.
Base Management – PCL: The first-year residency rotation schedule was strategically designed to align with the requirements of the NMLE. Key clinical departments, such as Respiratory Medicine, Cardiovascular Medicine, Neurological Medicine, General Surgery, Gynecology and Pediatrics, which represent major content areas of the NMLE, were prioritized within the rotation plan. This structured arrangement fostered a seamless integration of clinical practice, ongoing learning, and examination preparation, establishing a foundation for residents to acquire, comprehend, and apply theoretical knowledge within authentic clinical contexts while simultaneously preparing for the licensing board. Furthermore, this integrated framework supports the systematic implementation of Mentee-PAL group learning and Mentor-PBL teaching, which can be paced according to the overarching exam preparation timeline. Ultimately, this approach aimed to reinforce residents’ cognitive learning and knowledge retention through deliberate clinical exposure, embodying the principle of practice-enhanced cognitive learning (PCL).

### 2.3 Routine resident training teaching mode

Residents arranged their own review plans and were supervised by resident training mentors on a regular basis. The training base regularly organized simulation assessments and provided feedback to residents and training mentors. The training base arranged the resident rotation plan, and most of the first-year rotation took place at our emergency base.

### 2.4 Effectiveness evaluation

After residents had been recruited, the base organized a diagnostic exam. The score achieved in this exam was used as the baseline score for comparison. After each annual licensure examination, the examination scores and pass rates were used to compare the two groups. The primary outcome was licensure exam result, including pass rates and improvement based on the diagnostic exam. The secondary outcome was resident questionnaire.

Residents’ satisfaction with the improved teaching model: The questionnaire was developed specifically for this study based on satisfaction with base teaching and help in improving the six core competencies of residents (Professionalism, Patient Care, Communication & Collaboration, Integrated Clinical Proficiency, Teaching Competency and Lifelong Learning) (Supplementary Table 1). These six competencies represent precisely the overarching objective of the current residency training system in China. The options range from 1 to 10: 1–2 indicates very dissatisfied (no help); 3–4 indicates somewhat dissatisfied (some help); 5–6 indicates average satisfaction (somewhat helpful); 7–8 indicates fairly satisfied (helpful); 9–10 points indicates very satisfied (with a lot of help).

### 2.5 Statistical methods

Statistical analyses were performed using SPSS software (version 19.0). A dedicated statistical analysis plan was established prior to data examination to ensure methodological rigor. The specific tests employed were chosen based on the nature and distribution of the data, as detailed below:

#### 2.5.1 Descriptive statistics

Continuous variables were expressed as mean ± standard deviation (SD) if normally distributed, or as median with interquartile range (IQR) if non-normally distributed. Categorical variables were summarized as frequencies and percentages (*n*, %).

#### 2.5.2 Group comparisons

Continuous variables: For comparisons of continuous outcomes (e.g., NMLE scores, diagnostic exam scores, questionnaire ratings) between the pre-intervention and post-intervention groups, independent samples *t*-tests were used for data meeting assumptions of normality. The Mann–Whitney U test was applied for non-normally distributed data.

Categorical variables: Comparisons of categorical baseline characteristics (e.g., sex, CET certification) and the primary outcome of NMLE pass rates between the two groups were performed using the Chi-squared (*χ*^2^) test. Fisher’s exact test was used when expected cell counts were less than 5.

#### 2.5.3 Analysis of improvement and consistency

The magnitude of improvement from the diagnostic exam to the NMLE was calculated as a percentage and compared between groups using the Mann–Whitney U test. The consistency of pass/fail outcomes between the diagnostic examination and the NMLE within each cohort was assessed using McNemar’s test for paired nominal data.

A two-tailed *p*-value of less than 0.05 was considered statistically significant for all tests.

## 3 Results

### 3.1 General

This study included 24 first-year undergraduate residents before the implementation of MET model, and 22 residents after its implementation. There were no residents graduated from double-first-class universities. Since there is no national standardized exam for undergraduate graduation in China, resident physicians’ knowledge levels were indirectly assessed using College English Test (CET) results, stratified into three tiers: CET-6, CET-4, and no certificate. See Table 1 for more information.

**Table 1:** The general status of first-year residents before and after the implementation of MET model.

Characteristic	BEFORE	AFTER	*p* value
Total number of people	24	22	
Number of males (percentage)	15 (62.5%)	14 (63.6%)	>0.05
Median age	25 years (IQR 24–25)	25 years (IQR 24–25)	>0.05
College English test			
CET-6	4 (16.7%)	4 (18.2%)	>0.05
CET-4	12 (50%)	9 (40.9%)	>0.05
no certificate	8 (33.3%)	9 (40.9%)	>0.05
Number of residents passed the diagnostic exam (pass rate)	9 (37.5%)	4 (18.2%)	>0.05
Diagnostic exam scores	341 ± 35	300 ± 45	<0.05

### 3.2 Comparison of scores on the NMLE

See Table 2 for the passing status of the two groups of residents on the NMLE. Although the pre-implementation residents had higher scores on the diagnostic examination, their scores on the NMLE were significantly lower than post-implementation residents’ scores (388 ± 46 vs. 429 ± 41, *p* < 0.05). The passing rate for the licensure exam increased significantly after implementation (62.5% vs. 90.9%, *p* < 0.01). There was also a significant increase in the magnitude of improvement for licensure examination (14.6% ± 14.5% vs. 44.7% ± 16.4%, *p* < 0.05).

**Table 2:** NMLE results of first-year residents, before and after the implementation of MET model.

Outcome	BEFORE	AFTER	*p* value
Number of residents passed the NMLE (pass rate)	15 (62.5%)	20 (90.9%)	<0.01
NMLE scores	388 ± 46	429 ± 41	<0.01
Magnitude of improvement*	14.6% ± 14.5%,	44.7% ± 16.4%	<0.05
Consistency between the two exams #	*p* = 0.219	p < 0.01	

* The magnitude of improvement on the NMLE was calculated using the residents’ performance on the diagnostic examination as the baseline.

# The consistency between the two exams (diagnostic exam and the NMLE) was assessed with McNemar test.

Examinee performance was consistent across the two exams of pre-implementation residents. A McNemar’s test yielded a *p*-value of less than 0.01, indicating a significant discordance between the outcomes of the two examinations after the intervention, which indicates that the score improvement was attributable to the MET model.

### 3.3 Results of the resident questionnaires

**Figure 2:**
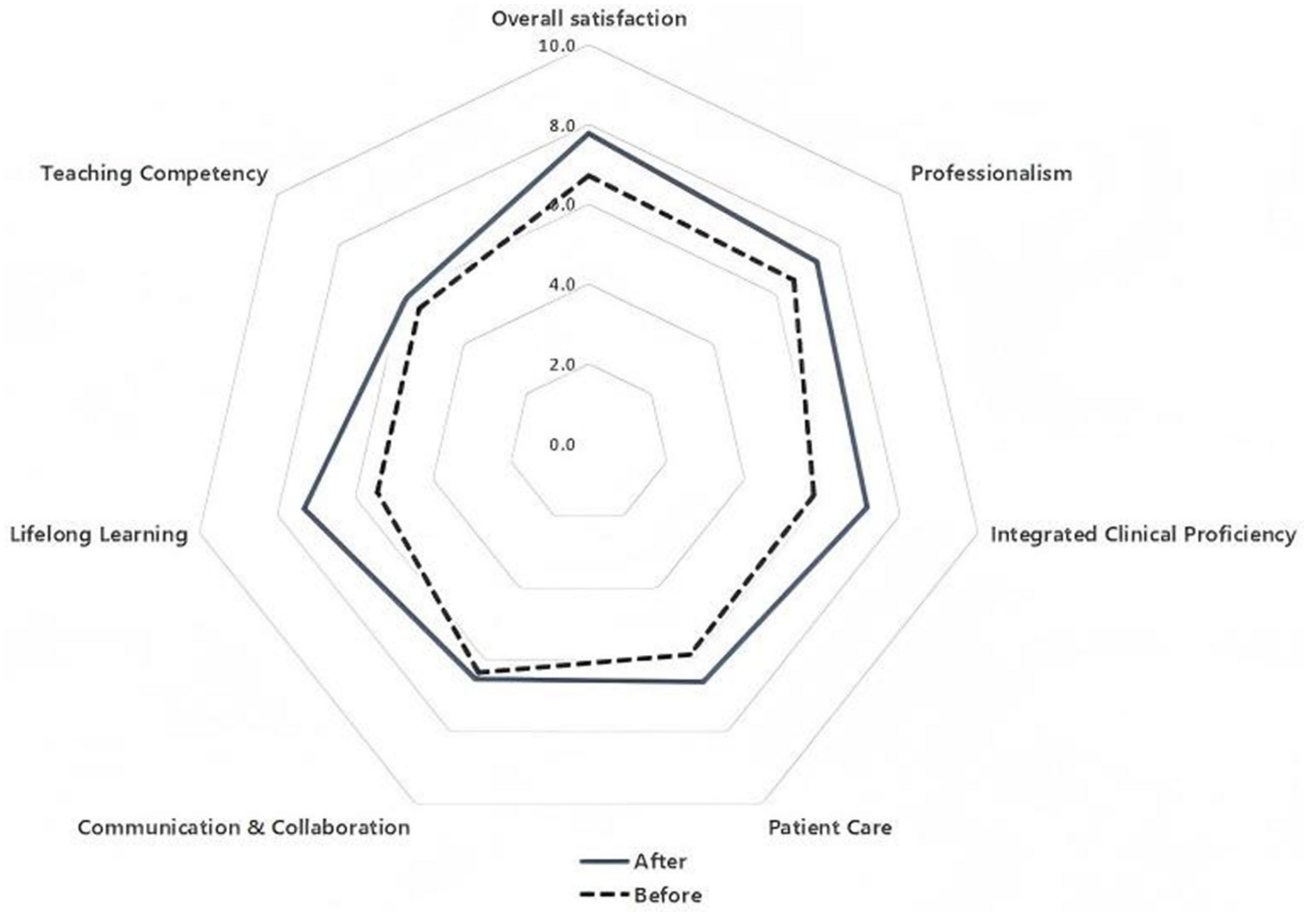
Radar chart showing the results of the questionnaires resident survey before and after the implementation of the MET model.

The results of the resident questionnaires on base teaching showed that overall satisfaction increased significantly after implementation (6.7 ± 0.7 vs. 7.8 ± 0.9, *p* < 0.05). After implementation, residents felt that base teaching had significantly improved their professionalism, professional competence, ability to manage patients, and ability to learn independently, as shown in Table 3 and Figure 2.

**Table 3:** Results of the resident questionnaires, conducted before and after the implementation of the MET model.

Competency/Satisfaction	BEFORE	AFTER	*p* value
Professionalism	6.6 ± 0.8	7.3 ± 0.8	<0.05
Integrated clinical proficiency	5.8 ± 0.8	7.2 ± 0.9	<0.01
Patient care	5.9 ± 0.9	6.6 ± 0.7	<0.05
Communication & collaboration	6.4 ± 0.8	6.5 ± 0.6	>0.05
Lifelong learning	5.4 ± 0.6	7.3 ± 0.9	<0.01
Teaching competency	5.4 ± 0.8	5.8 ± 0.8	>0.05
Overall satisfaction	6.7 ± 0.7	7.8 ± 0.9	<0.01

## 4 Discussion

Many residency training bases have received unsatisfactory scores on the NMLE (10), and some have conducted relevant researches and summarized teaching methods and experiences. These studies tended to focus more on directly improving residents’ performance on licensing examinations—for instance, by predicting their exam results, increasing theoretical instruction and clinical skills training, urging residents to brush up exam questions, and organizing repeated simulation exams (6, 7). Such measures essentially function as an intensive training program tailored specifically for the exam. While they may help boost test scores to some extent, they contribute limited support to residents’ lifelong learning and long-term career development. We focused more on the deeper reasons for residents’ unsatisfactory performance, particularly among those who have graduated from non-prestigious institutions, i.e., a lack of metacognitive skills. This can trigger a cascade of effects on resident physicians, impacting their learning motivation, approaches, and ultimately, their learning effectiveness (11). The identified challenges are threefold. First, intrinsic motivation is often inadequate, with residents failing to formulate or consistently execute long-term learning plans. Second, study methods are suboptimal, lacking the depth of analysis and synthesis required to build an integrated knowledge framework, which consequently impedes a profound understanding of theoretical concepts. Third, a significant gap exists between clinical practice and learning, as residents seldom consciously leverage clinical encounters to reinforce theoretical knowledge. It is therefore imperative that our pedagogical reforms focus on enhancing metacognition among residents.

PAL is a form of supplemental instruction often implemented in medical schools, particularly during the clinical phase of students’ training (12). PAL has been shown to significantly impact medical students’ self-confidence and self-perception, as well as their practical skills and clinical competence (13). Peer learning serves to motivate students and develop metacognitive skills through distinct yet complementary mechanisms. The motivational aspect, driven by peer effects, upward social comparisons, and group belonging, fosters increased self-efficacy and interest, shifting the driver of learning from external demands to internal needs. Concurrently, it provides a framework for shared metacognitive regulation through tasks requiring explanation, peer feedback, and reflection. This framework compels students to articulate and refine their cognitive processes, thereby enhancing their metacognitive skills. PAL is widely used in medical schools in the United Kingdom and the United States, and studies have demonstrated a positive correlation between its use and test scores (14).

While PAL demonstrates limitations in cultivating higher-order problem-solving abilities (15), its integration with PBL addresses this deficit through structured cognitive scaffolding. PBL is derived from the constructivist teaching philosophy, which has been shown to effectively improve learners’ critical thinking, creativity, and problem-solving skills (16). A key principle of constructivist teaching is the belief that mental models of learning and individuals are constantly evolving, with a focus on continuous learning and improvement in practice (17). The pedagogical strengths of PBL are twofold. Firstly, it stimulates intrinsic motivation by empowering students with autonomy in problem-solving, fostering a sense of belonging through collaboration and developing competence by overcoming complex challenges. This approach effectively addresses the core psychological needs outlined in Self-Determination Theory. Secondly, instructors in PBL enhance metacognition. By posing guiding questions and facilitating discussions, they prompt students to plan, monitor and evaluate their learning, making internal cognitive processes explicit. In this role as an “expert learner,” the instructor provides scaffolding that models advanced reasoning, thereby enabling students to internalize metacognitive strategies.

Some studies have also shown that medical students who participate in problem-based learning (PBL) are more interested in and in need of clinical practice sessions (18). Therefore, PCL is a powerful complement to PBL. We adapted the original rotation program to align with the learning objectives of the first year of residency. The first-year rotation plan was restructured around the key content domains of the NMLE, with the explicit aim of integrating the examination preparation timeline directly into the clinical schedule. According to Kolb’s model, learning is a cycle starting with concrete experience, progressing through reflective observation and abstract conceptualization, and culminating in active experimentation. Clinical practice is the ideal manifestation of this cycle. By anchoring abstract knowledge in specific clinical situations, practice conditionalizes knowledge—teaching the learner when and why to use it—which directly addresses the emphasis on clinical thinking and application in modern licensing exams.

Based on the principles of metacognitive enhancement, we integrated three key dimensions—residents, mentor, and base management—employing PAL, PBL and PCL methodologies, respectively. These approaches complemented and reinforced each other, with the enhancement of residents’ metacognitive abilities serving as the central focus to maximize instructional effectiveness. Study results demonstrated that our MET model significantly improved residents’ pass rates and scores on the NMLE. Furthermore, survey responses from the residents indicated that the MET model enhanced multiple competencies (professionalism, professional competence, ability to manage patients, and ability to learn independently), and was well received.

Despite the promising results, this study has several limitations. First, the effectiveness of the MET model is partially dependent on the quality and engagement of peers and mentors. Variability in the facilitating skills of peer leaders and the commitment and teaching proficiency of mentors could lead to inconsistent learning experiences and outcomes across different PAL and PBL groups. Second, the quasi-experimental design, comparing two historical cohorts, carries an inherent risk of selection bias. Although we used a diagnostic examination to establish a baseline and found that the pre-intervention cohort actually had a higher baseline score, we cannot completely rule out the influence of unmeasured confounders. Additionally, this was a single-center study with a relatively small sample size, which may limit the generalizability of the findings. The model was implemented within a single specialty (Emergency Medicine) at a tertiary academic center, and its efficacy in other disciplines or healthcare settings remains to be investigated. Furthermore, the model showed limited impact on improving communication and teaching skills, suggesting that these competencies require more targeted instructional. We look forward to conducting further multi-center controlled studies in the future to validate and expand upon these findings.

Our results indicate that implementing the MET model in the standardized residency training base effectively enhances residents’ metacognitive skills, leading to improved performance on the NMLE. This outcome not only facilitates the achievement of standardized training objectives but also lays a solid foundation for residents’ continuous professional development (19).

## 5 Conclusion

To improve the pass rate of the NMLE and enhance the overall outcomes of standardized residency training for undergraduate residents, we developed a novel and systematic teaching methodology focused on enhancing residents’ metacognitive learning abilities, known as the Metacognitive Enhancement Triad (MET) model. Although the sample size was small, we witnessed the success of the MET model through an increased pass rate in the NMLE. In spite of the improvements, there are still some limits, so we look forward to conducting further multi-center controlled studies in the future, and we hope that our MET model can bring benefits to other training bases or areas of medical education.

## Data Availability

The raw data supporting the conclusions of this article will be made available by the authors, without undue reservation.
