# Supplementary material for: The Metacognitive Enhancement Triad model enhances learning outcomes in first-year emergency medicine residents
Source: Front Med (Lausanne). 2025 Nov 17;12:1720071. doi: 10.3389/fmed.2025.1720071 (PMC12667245; doi:10.3389/fmed.2025.1720071)
Supplement: Supplementary file 1 [file Table_1.docx]

Supplementary Table 1. Resident Teaching Satisfaction Survey

|  | Very Dissatisfied / Not helpful at all | | Somewhat Dissatisfied / Slightly helpful | | Neutral / Moderately helpful | | Satisfied / Helpful | | Very Satisfied / Extremely helpful | |
| --- | --- | --- | --- | --- | --- | --- | --- | --- | --- | --- |
|  | 1 | 2 | 3 | 4 | 5 | 6 | 7 | 8 | 9 | 10 |
| To what extent has the base contributed to the development of your professionalism? |  |  |  |  |  |  |  |  |  |  |
| To what extent has the base contributed to the development of your integrated clinical proficiency? |  |  |  |  |  |  |  |  |  |  |
| To what extent has the base contributed to the development of your patient care skills? |  |  |  |  |  |  |  |  |  |  |
| To what extent has the base contributed to the development of your communication and collaboration skills? |  |  |  |  |  |  |  |  |  |  |
| To what extent has the base contributed to the development of your lifelong learning ability? |  |  |  |  |  |  |  |  |  |  |
| To what extent has the base contributed to the development of your teaching competency? |  |  |  |  |  |  |  |  |  |  |
| What is your overall satisfaction with base teaching |  |  |  |  |  |  |  |  |  |  |
